# Effects of motivations in marine protected areas: The case of Galápagos Islands

**DOI:** 10.1371/journal.pone.0293480

**Published:** 2023-11-02

**Authors:** Mauricio Carvache-Franco, Wilmer Carvache-Franco, Ana Beatriz Hernández-Lara, Orly Carvache-Franco

**Affiliations:** 1 Universidad Espíritu Santo, Samborondón, Ecuador; 2 Facultad de Ciencias Sociales y Humanísticas, Escuela Superior Politécnica del Litoral, ESPOL, Guayaquil, Ecuador; 3 Departament de Gestió d’Empreses, Universitat Rovira i Virgili, Reus, Spain; 4 Facultad de Economía y Empresa, Universidad Católica de Santiago de Guayaquil, Guayaquil, Ecuador; Fiji National University, FIJI

## Abstract

This research proposes: (i) analyze push and pull motivations in insular marine protected areas, and (ii) determine their effects on tourists’ satisfaction, positive recommendations and loyalty in terms of returning. The study was conducted in the Galápagos Islands in Ecuador; a renowned marine protected area with a National Park being a Natural Heritage Site of Humanity by UNESCO. An exploratory (EFA) and confirmatory factor analysis (CFA), followed by a stepwise multiple regression analysis applied on a sample of 407 tourists in the Galápagos Islands showed four push and pull motivational factors in this destination: "passive marine," "active marine," "novelty and escape," and "social relations". "Passive marine" and "social relations" were the most prominent factors influencing tourist satisfaction and return intentions. Moreover, "passive marine" and "novelty and escape " highly influenced visitors’ intention to recommend and give positive feedback about this place. These findings constitute action guides for the adequate management of marine protected areas.

## 1. Introduction

National parks with coastal and marine resources are forced to find alternatives to preserve the environment and develop diverse sustainable tourism experiences that complement and enrich the classic sun and beach tourism. Orams and Lueck [1] identified some activities, like those involving nature, wildlife, wellness and sport, commonly practiced in coastal tourism. Orams and Lueck [2] also emphasized the relationship between marine tourism and coastal tourism, given that coastal sites are where normally marine activities begin and end [2]. Recently, destinations with coastal resources give their visitors a wide range of alternative activities for their stay and recreation that enrich their tourism experience [3].

Tourism demand studies on coastal and marine destinations have addressed especially visitors’ motivations as a determinant factor that influences this demand [4, 5]. Motivations refer to the needs that make people have the desire to intervene in tourist activities [6], being the push-pull theory of motivation [7, 8] one of the central theoretical frameworks used to explain the reasons for tourist trips and activities [9].

According to Bayih and Singh [10], the motivations that push and attract have been important predictors of tourists’ activities, and exert a high positive predominance in the behavior and satisfaction of visitors towards specific tourists activities and destinations [11].

Pull motivations refer to specific lure and characteristics of regions that may attract tourists based on the things that the place offers [12]. Push factors, on the other hand, entail the personal desires of tourists, and are frequently considered the prominent paradigm, over the pull factors, due to their influence on tourists’ search internal processes [13].

The Galápagos Islands in Ecuador, with their National Park and Marine Reserve, constitute a protected marine area, and received the nomination of World Heritage, granted by UNESCO in 1978. The location of the Archipelago, in the concurrence of marine currents, provokes the existence of specific marine flora and fauna. Its endemic species attract visitors who carry out activities such as snorkeling, surfing, diving, watching marine fauna, and enjoying themselves on its beaches. The unique characteristics of this destination explain the different awards and recognitions obtained in the last years.

The Galápagos Islands are an ideal destination for tourism demand studies because of their unique coastal and marines characteristics and resources, and endemic natural species. Considering that motivations can predict tourist behavior in this typology of tourism, it is crucial to deepen their study to generate management plans for the sustainable preservation of these sites. However, to the authors’ best knowledge, push and pull motivations have not been considered by previous research to predict tourists’ satisfaction and the future behavior of visitors in coastal and marine sites of insular protected areas. Accordingly, this research carried out in the Galápagos Archipelago has as objectives: (i) to analyze tourists’ motivations (push/pull) and (ii) to determine their influence on satisfaction, positive recommendations and loyalty in terms of returning.

We expect to contribute to marketing academic literature of coastal tourist destinations in protected areas. It would offer tourist managers action guides to create sustainable development plans and would give service providers accurate knowledge of the demand attracted to this kind of destination.

## 2. Theoretical framework

### 2.1. Motivations in marine and coastal destinations

Tourism literature is plenty of studies using process models to predict tourists’ behavior, including demand [7, 14, 15].

Kassean and Gassita [16] studied the push and pull motivators that explain the selection of Mauritius as a holiday destination. The authors found that the main driving forces were rest and relaxation, then escape, nostalgia, novelty and the social dimension. Among the pull motivations, they mentioned Mauritius’ climate, beaches, landscape, nature elements, atmosphere and authentic culture.

Yiamjanya and Wongleedee [17] analyzed motivational dimensions to visit Thailand. Among the push factors, they highlighted new experiences, relaxation, culture, novelty, joy, and adventure. Among the attraction factors, traditional markets, weather and food were emphazised. Likewise, Jeong [18] analyzed the association between motivations and loyalty of marine tourists. Getting out of the routine and living new experiences occupied a notable position among the push forces, while passive and active marine activities were the most salient pull motivators.

In the same way, the motivations of British tourists visiting Phuket, Thailand, were analyzed [19]. Among the push motivations emerged: relaxation, fun, and escape. The main attractions were related to natural elements, like landscape and beaches, and the hospitality and friendliness of locals. In New Zealand, Oceania, Fieger et al. [20] found nine types of international tourists in terms of their motivations. The most relevant were the visitors attracted by the nature activities that take place in New Zealand, followed by those who love adventure. Wen and Huang [21] studied the motivational factors of Chinese tourists in Cuba, and found that unique experiences on the one hand, and the attractions offered by the destination, on the other, emerged as the main push and pull factors respectively. Socialist nostalgia was the main predictor for visitors to return. Carvache-Franco et al. [22] found in a costal destination like Salinas (Ecuador), that “escape and novelty” and “experiences and knowledge” were two relevant push forces, while “active” and “passive” marine were the most outstanding pull motivators. Finally, Güzel et al. [23] analyzed in a recent study visitors’ push travel motivations in Antalya (Turkey), considering especially the role of emotions. Escape, relaxation, curiosity, active life and sport, travel bragging, and extravagance were emphasized as the most salient push motivators.

In summary, previous studies suggest that the motivations to visit different coastal destinations may vary according to their characteristic and the tourist activities that they offer. However, recurring common motivating factors can also be found, such as socialization, escape, and novelty. It is necessary then to carry out this type of research in marine protected areas, to better understand the basis of the demand in these specific coastal destinations, taking into account their particularities.

### 2.2. Motivations effects on loyalty and satisfaction

Motivations may influence visitors’ satisfaction and loyalty [24]. For example, Assaker, Vinzi and O’Connor [25] stated the positive association between novelty and visitors’ satisfaction. Likewise, several studies found a positive effect of novelty on the intention to return [26–29]. More specifically, Alipour et al. [30] in a sun and beach place found that tourist attitudes towards sun and sea positively influences tourists’ visits intentions and their word-of-mouth. Carvache-Franco et al. [22] also argued out the factors that predict tourists’ loyalty in Salinas (Ecuador), stating the role of “escape and novelty” and “passive marine”, as the most relevant predictors of the intention to return to and recommend this coastal marine tourist place.

On the other hand, satisfaction and loyalty are also connected. As a matter of fact, several studies have highlighted satisfaction as a main predictor of tourists’ loyalty [24, 25], and some of them have also emphazised the interconnection of the three variables, motivations, satisfaction and loyalty. For example, Pestana et al. [31] found that satisfaction mediates the relationships between motivations, emotions and future behavior, like intention of revisiting, of older travelers visiting Lisbon, Portugal. Rice and Khanin [32] identified that repeated visits to tourist destinations in the United States were influenced by satisfaction and motivations (push/pull), being the influence of push factors the strongest, and acting both as mediator variables. Likewise, Fianto [33] argued that the quality of service and brand experience significantly affected visitors’ intention to return to a place, acting visitors’ satisfaction at the same time as a mediator of the relationship between market experience and the revisit intention.

To sum up, among the main motivations, escape and novelty play a key role predicting tourist behavior, but more evidence is necessary in marine protected areas to reveal the most influent tourists’ motivators.

## 3. Area of study

The Galápagos Islands or Galápagos Archipelago, in the eastern part of the Pacific Ocean, is a province of Ecuador, 972 km from the continental coasts. Seven main islands constitute the Archipelago. Tourists arrive throughout the year and from all over the world. In 2022, the Galápagos Islands received 267,688 tourists [34].

The Archipelago includes the National Park and Marine Reserve, both protected areas. The Reserve is considered an important marine life sanctuary, with seamounts and nutrient outcrops caused by the Cromwell Undercurrent. These areas are home to sea lions, giant tortoises, marine and land iguanas, seabirds, and other species. Tourists can visit the Sleeping Lion and the Junco lagoon, a freshwater lake home of exotic birds; Lobería Beach, ideal for tourist activities, with rocky and sandy trails; and Mann Beach, formed by white sand and broken shells with transparent waters for snorkelling. Other relevant attractions for tourists are the white sand beaches of Turtle Bay (nesting site of the black turtle), the crystal clear waters and gentle waves of the Garrapatero beach, or the Charles Darwin Research Station.

Within the field of demand studies in tourism, the Galápagos Islands deserve special attention due to the need of protection of their unique biodiversity, endemic natural species, and marine resources. Therefore, studying the characteristics of its tourist demand is relevant to protect the destination and guarantee its sustainability.

## 4. Methodology

The data collection of this research was performed through a questionnaire based on previous research on motivational factors of coastal places. The questionnaire consisted of 16 questions divided into three sections. One part, composed of 12 items, was adapted from Jeong [18], and applied to analyze the tourists’ motivations. The second section, composed of 4 items based on the study of Kim and Park [35], referred to satisfaction and loyalty. These two sections used a 5-point Likert scale (where 1 was "not very important" and 5 was "very important"). Finally, there was also a section to capture the sociodemographic characteristics of tourists, composed by closed questions adapted from the research by [36].

The study population was composed of domestic and foreign tourists aged 18 years or over, who enjoyed the marine protected areas in Mann Beach (San Cristobal Island) during January and February 2019. The data were gathered, using the convenience methodology to choose the sample, with self-administered questionnaires. The distribution of these questionnaires was done by trained professionals residing in the location. They approached the tourists when they were resting freely.

The valid sample reached 407 responses (margin of error of +/- 5%, confidence level of 95%, and variation of 50%). This sample size was appropriate because a larger sample size does not reduce the sampling error in the same proportion.

## 5. Results

In the next sections, we explain the descriptive statistics used to identify the sample’s sociodemographic profile. Afterward, we present the push and pull factors in the Galápagos Islands obtained through factor analyses, first exploratory (EFA) and then confirmatory (CFA). Finally, the influence of the motivational factors on tourists’ satisfaction and loyalty is analyzed through stepwise multiple regression analysis.

### 5.1. Sociodemographic aspects

The sample of this study was composed of foreign and domestic tourists (84.3% and 15.7%, respectively). Most of the surveyed visitors came from Europe (38.6%) and North America (23.1%). In relation to gender, the sample was quite balanced, with 55.8% of men and 44.2% of women, most of them single (59.2%). The highest percentage belongs to an age between 21 and 30 years old (42.15%). In terms of education, 62.9% of the tourists had university studies. Most of them were private employees (30.7%). Table 1 summarizes these results.

**Table 1:** Sociodemographic features.

Demographic Variables	Levels	N = 407	%
Origin	Domestic	64	15.7
	Foreign	343	84.3
Gender	Man	179	44.2
	Woman	227	55.8
Marital status	Single	241	59.2
	Married	122	30
	Other	36	8.8
Age	<20 years old	23	5.7
	21–30	171	42.1
	31–40	142	35
	41–50	44	10.8
	61–60	18	4.4
	>60 years old	8	2
Education	Primary	11	2.7
	Secondary	52	12.8
	University	256	62.9
	Postgraduate/Master/Ph.D.	88	21.6
Professional activity	Student	105	25.8
	Researcher / scientist	19	4.7
	Businessman	64	15.7
	Private Employee	125	30.7
	Public Employee	58	14.3
	Retired	12	2.9
	unemployed	8	2
	Other	16	3.9

### 5.2. Exploratory Factor Analysis (EFA)

Four dimensions were identified applying EFA, with principal components analysis and varimax rotation. Two were labeled as push motivations and the other two as pull motivations. The four factors captured 65.71% of the total variance, and their items showed internal consistency with a Cronbach’s alpha of 0.8, ranging for each factor between 0.759 and 0.578. Table 2 displays the results.

**Table 2:** EFA results.

Variables	Components				Factors
	1	2	3	4	
Clean water and fresh air	0.853				Pasive Marine (Pull)
Activities at the beach	0.792				
Scenic beauty of beaches	0.619				
Cruises		0.781			Active Marine (Pull)
Marine sports		0.731			
Swimming		0.581			
To escape from routine			0.746		Novelty and escape (Push)
To recharge spirits			0.707		
To experience novelty			0.658		
To introspect			0.574		
To enjoy with local population				0.887	Social relations (Push)
To share with locals				0.872	
Cronbach’s α	0.759	0.623	0.578	0.753	
KMO	0.81				
Eigen-values	4.27	1.42	1.12	1.08	
Variance explained (%)	35.55	11.83	9.3	9.02	
Total variance explained (%)	65.71				
Bartlett’s Test of Sphericity	Chi-square = 1533.80 sig. = 0.000				

As shown in Table 2, the first dimension, called "passive marine", was made up of tourists who want to enjoy the beach, appreciating its beaty, fresch air and clear water. The second factor refers to visitors motivated to parcipate on cruises and doing marine sports, being labeled “active marine”. The third dimension, “novelty and escape” included tourists motivated to change their routine and spirit, who appreciate novely and introspection. The last factor, “social relations”, included tourists motivated to share and enjoy with local population.

### 5.3. Confirmatory Factor Analysis (CFA)

**Fig 1:**
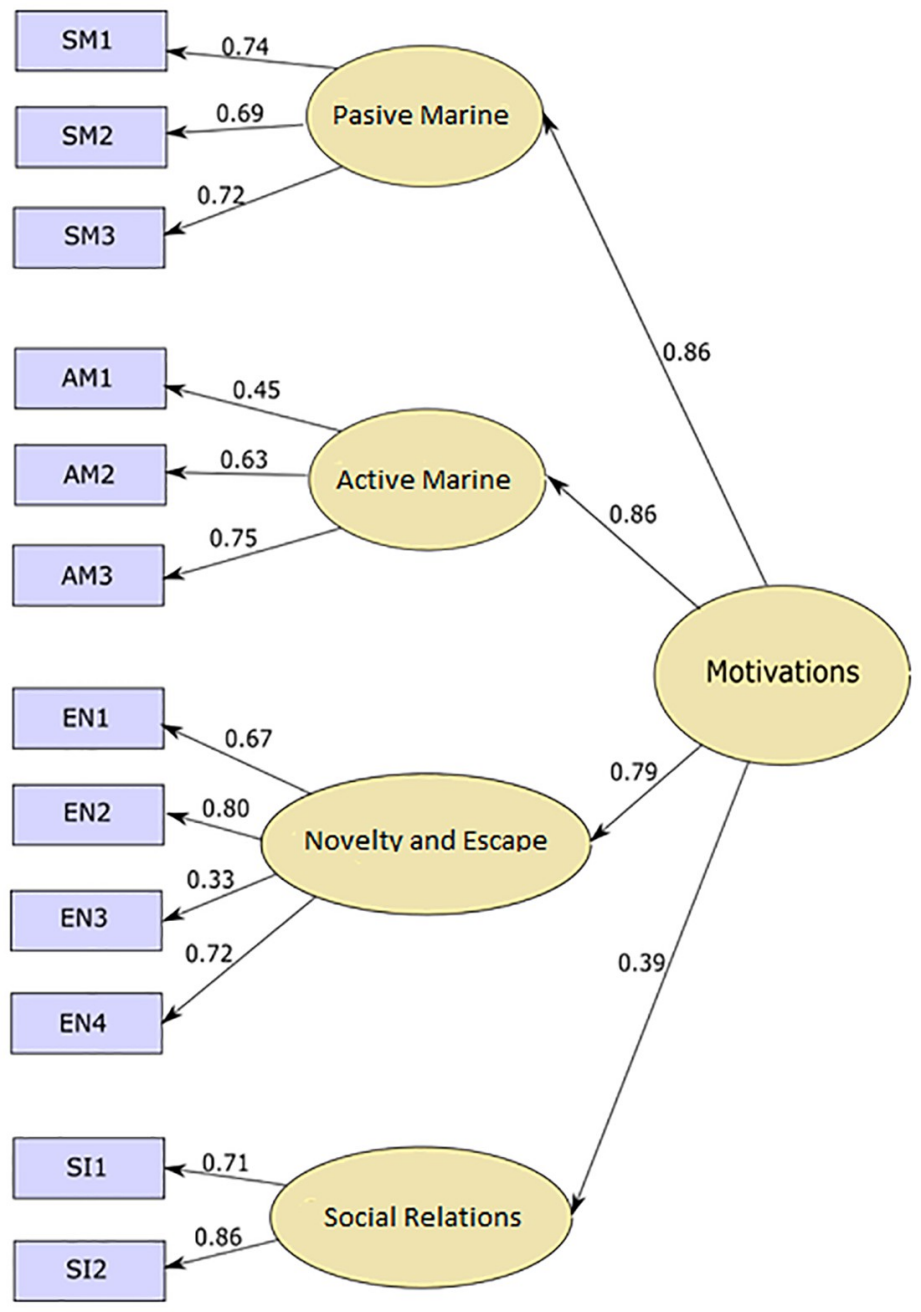
Measurement model.

In this study we applied a CFA using maximum likelihood, which assured us of the reliability of the measurement model (See Fig 1).

Tests conducted to check the model fit showed acceptable values, with a χ2 = 3.923 (p < .000), a comparative fit index (CFI) = 0.901, a normed-fit index (NFI) = 0.874, and a root mean square error of approximation (RMSEA) = 0.08. To sup up, the statistics accomplished the recommended levels to guarantee the fit of the proposed model [37–42], meaning that the model of this study was theoretically meaningful and had statistically acceptance [43–45].

### 5.4. The relationship between motivations and satisfaction

A backward stepwise regression procedure was used in this study to analyse the relationship between motivations and tourists’ satisfaction and loyalty dimensions in a marine and coastal destination. This method allows all explanatory variables to be entered at an early stage, and in the next stages eliminates the variables with lowest partial correlation with the dependent variable [46]. Table 3 displays the results referred to motivations and satisfaction.

**Table 3:** Relationships between motivations and tourists’ satisfaction.

Factors	Standardized Beta	t	Sig.
Passive marine	0.341	7.323	0.000
Social relations	0.101	2.164	0.031
(Constant)		97.31	0.000
Adj. R2	0.122		
F statistic	29.156		
Sig.	0.000		

All the models consistency was observed through the significance of the F test (p <0.05). In addition, no multicollinearity problems were detected between the independent variables in all the models.

In the specific case of satisfaction, Table 3 shows two motivational dimensions with relationship with tourist satisfaction which explained 12.2% of the total variance. The "passive marine" attraction factor had the strongest relationship with tourists’ satisfaction in the chosen destination (β = 0.341, p <0.05). The push dimension "social relations" was also associated to tourists’ satisfaction (β = 0.101, p <0.05).

### 5.5. The relationship between motivations and the intention to return

Table 4 shows the results of the regression analysis for the relationship between motivations and intention to return of tourists.

**Table 4:** Relationships between motivations and intention to return.

Factors	Standardized Beta	T	Sig.
Passive marine	0.237	4.956	0.000
Social relations	0.133	2.784	0.006
(Constant)		63.102	0.000
Adj. R2	0.069		
F statistic	16.158		
Sig.	0.000		

Again, as shown in Table 4, two motivational factors influenced return intentions, representing 6.9% of the variance. "Passive marine" was the most significant attractor for tourists’ intentions to return to the chosen destination (β = 0.237, p <0.05), followed by the push of “social relations” (β = 0.133, p <0.05).

### 5.6. The relationship between motivations and destination recommendation

Table 5 illustrates the results on the association between motivations and the intention of tourist to recommend the destination.

**Table 5:** Relationships between motivations and recommendations.

Factors	Standardized Beta	T	Sig.
Passive marine	0.404	8.936	0.000
Novelty and escape	0.104	2.307	0.022
(Constant)		96.115	0.000
Adj. R2	0.170		
F statistic	42.590		
Sig.	0.000		

Based on the findings (Table 5), two motivators had a significant relationship with the intentions to recommend the destination, “passive marine" attraction factor (β = 0.404, p <0.05) and “novelty and escape” (β = 0.104, p <0.05), accounting for 17% of the variance.

### 5.7. The relationship between motivations and positive feedback of tourists

Table 6 exhibits the motivators related to the positive feedback provided by tourists.

**Table 6:** Relationships between motivations and positive feedback.

Factors	Standardized Beta	T	Sig.
Passive marine	0.327	6.984	0.000
Novelty and escape	0.102	2.188	0.029
(Constant)		108.917	0.000
Adj. R2	0.113		
F statistic	26.785		
Sig.	0.000		

As seen in Table 6, two motivational factors significantly influenced tourists’ intentions to speak positively about the destination, explaining 11.3% of the total variance, “static marine” (β = 0.327, p <0.05) and “novelty and escape” (β = 0.102, p <0.05).

## 6. Discussion

This study had as first objective to analyze tourists’ motivators (push/pull) in insular marine protected areas as a type of marine and coastal tourism. The results found two push and two pull motivations in the Galápagos Archipelago.

"Novelty and escape" and "social relations" emerge as prominent push motivations, confirming the findings of previous studies. For example, Kassean and Gassita’s [16] found escape, novelty, and social interaction, among other dimensions, as relevant motivators. Likewise, Sastre and Phakdee-Auksorn [19] found rest and relax, fun, and the environment, as push motivations, highlighting in these factors some similar characteristics to our dimensions of “novelty and escape”. This research also supports Jeong’s findings [18] and those of Carvache-Franco et al. [22], regarding the “escape and novelty” dimension. Concerning the pull motivations, the present study confirms the salient role of "active marine" and "passive marine", also found by Jeong [18] and Carvache-Franco et al. [22]. The main contribution of the present research is the identification of the push factor, labeled as “social relations”, as an important attractor in the specific case of insular marine protected areas as part of coastal and marine destinations.

Our second objective was to determine the motivations related to tourists’ satisfaction, their intention to return, recommend and share positive feedback. The results indicate that "novelty and escape" is the most significant push factor related to tourists’ intention to speak positively about this destination and recommend it so other tourists may visit as well. These findings agree with previous research on the topic. For Huyen and Nghi [29], novelty-seeking also positively impacted loyalty and satisfaction of visitors. Similarly, Wen and Huang’s [21] found that the search for unique experiences, specific attractions, and socialist nostalgia, predicted the revisit intention in the case of Chinese cigar tourists visiting Cuba. Other previous investigations had also demonstrated that novelty had a positive influence on future intentions to return to specific destinations and tourists’ satisfaction [25]. The main novelty of our research is that we also found other push motivation, “social relations” significantly related to the intention to return and the satisfaction of tourists in our chosen destination. In addition, the motivational pull dimension "passive marine" emerges as a relevant factor related to satisfaction and loyalty in its multiple dimensions. Although this research identified two pull factors, only the "static marine" was proven to significantly influence tourists’ satisfaction and loyalty to the Galápagos Islands. Among these factors, its natural resources, beauty, beaches, and tranquility stood out over other more active activities and sports. These findings demonstrate that pull factors were more important than the push ones regarding satisfaction and loyalty, constituting a relevant contribution that emerges from the results.

All in all, this study makes a relevant contribution to previous related research, pointing out a new push dimension, named “social relations”, in coastal and marine tourist destinations. This new push motivational dimension was the one with the greatest impact on the tourists satisfacction and their intention to return. We think that it is important to continue studying the impact of this new dimension to generalize and confirm the robustness of this result in this kind of destinations. These findings were not affected by the impacts of external factors such as the COVID 19 pandemic, since push and pull motivations form positive (not negative) impulses and attractions of tourists to travel to a tourist destination.

## 7. Conclusions

From the results of this study focused on tourism in marine protected areas, it could be concluded that the beauty of the beach, the activities that tousrist can carry out there, enjoying the clear waters and fresh air, as well as sharing experiences with locals, should characterised the tourist offer in protected marine and coastal destinations. Similarly, escaping from everyday life and experiencing novelty are relevant factors that positively influence tourists’ intention to recommend and speak favorably about these places.

These findings provide some theoretical implications. Particularly, "social relations" appears as a new driving factor in insular marine protected areas. Likewise, "passive marine", "social relations", and "novelty and escape" are prominent motivational factors that explain the satisfaction of tourists and their intentions to return, recommend and give positive feedback.

The study also provides practical implications that imply that tourism companies and other kind of tourist institutions can obtain information on the motivations related to the intentions of visitors to return and recommend this kind of tourist destination. These results may guide tourism institutions in an efficient planification of their marketing strategies to increase the intentions of tourists to revisit these destinations. Some social implications also emerge from the findings, related to the relevance of the interaction with local population as an attractor of tourists in these specific destinations, which entails sustainable principles, in their different spheres, economic, social and environmental.

Among the limitations, it is important to mention the temporality of this study, given that the demand can vary and fluctuate over time, a longitudinal research with data in different periods should help to avoid potential biases. However, this type of study is carried out in a short period of time, due to the length of time tourists spend in the destination. As a future research line, it would be advisable to develop studies on tourist products that foster push and pull tourists’ motivations to analyse their effect on loyalty and satisfaction.

## Supporting information

S1 Data (SAV)

## References

[1] Orams M., Lueck M. “Coastal tourism.” Jafari J, Xiao H, editors. Encyclopedia of Tourism. Springer International Publishing.; 2016. 157–158 p.

[2] Orams M., Lueck M. “Marine tourism.” Jafari J, Xiao H, editors. Encyclopedia of Tourism. Springer International Publishing.; 2016. 585–586 p.

[3] Carvache-Franco W, Carvache-Franco M, Carvache-Franco O, Hernández-Lara AB. Motivation and segmentation of the demand for coastal and marine destinations. *Tour*. *Manag*. *Perspect*. 2020;34:100661. doi: 10.1016/j.tmp.2020.100661

[4] Jacobsen JKS, Antonson H. Motivational segments for trips along the high coast byway of Sweden: A study of local leisure excursions and domestic holidaymaking. *Scand*. *J*. *Hosp*. *Tour*. 2017;17(2):177–93.

[5] Pesonen JA. Segmentation of rural tourists: Combining push and pull motivations. *Tour*. *Hosp*. *Manag*. 2012;18(1):69–82. https://hrcak.srce.hr/83824

[6] Swanson KK, Horridge PE. Travel motivations as souvenir purchase indicators. *Tour*. *Manag*. 2006;27(4):671–83. doi: 10.1016/j.tourman.2005.03.001

[7] Crompton JL. Motivations for pleasure vacation. *Ann*. *Tour*. *Res*. 1979;6(4):408–24. doi: 10.1016/0160-7383(79)90004-5

[8] Dann GMS. Anomie, ego-enhancement and tourism. *Ann*. *Tour*. *Res*. 1977;4(4):184–94. doi: 10.1016/0160-7383(77)90037-8

[9] Otoo FE, Kim S. Analysis of studies on the travel motivations of senior tourists from 1980 to 2017: Progress and future directions. *Curr*. *Issues Tour*. 2020;23(4):393–417. doi: 10.1080/13683500.2018.1540560

[10] Bayih BE, Singh A. Exploring domestic tourism in Ethiopia: trends, prospects, promotional marketing, and challenges. *Int*. *J*. *Recent Technol*. *Eng*. (IJRTE). 2020;8(6):2675–88. doi: 10.35940/ijrte.F8215.03862

[11] Khuong MN, Ha HTT. The influences of push and pull factors on the international leisure tourists’ return intention to Ho Chi Minh City, Vietnam—a mediation analysis of destination satisfaction. *Int*. *J*. *Trade*, *Econ*. *Finance*. 2014;5(6):490. doi: 10.7763/IJTEF.2014.V5.421

[12] Bansal H, Eiselt HA. Exploratory research of tourist motivations and planning. *Tour*. *Manag*. 2004;25(3):387–96. doi: 10.1016/S0261-5177(03)00135-3

[13] Armario EM. Tourist satisfaction: an analysis of its antecedents. In: Universidad, Sociedad y Mercados Globales. *Spanish Association Bus Manag*. *Econ*. (AEDEM); 2008. p. 367–82. https://dialnet.unirioja.es/servlet/articulo?codigo=2751774

[14] Prayag G, Hosany S. When Middle East meets West: Understanding the motives and perceptions of young tourists from United Arab Emirates. *Tour*. *Manag*. 2014;40:35–45. doi: 10.1016/j.tourman.2013.05.003

[15] Zhang Y, Peng Y. Understanding travel motivations of Chinese tourists visiting Cairns, Australia. *J*. *Hosp*. *Tour*. *Manag*. 2014;21:44–53. doi: 10.1016/j.jhtm.2014.07.001

[16] Kassean H, Gassita R. Exploring tourists push and pull motivations to visit Mauritius as a holiday destination. *Afr*. *J*. *Hosp*. 2013;2(3):39–56. http://www.ajhtl.com/uploads/7/1/6/3/7163688/article_5_2013.pdf

[17] Yiamjanya S, Wongleedee K. International tourists’ travel motivation by push-pull factors and the decision making for selecting Thailand as destination choice. *Int*. *J*. *Soc*. *Educ*. *Econ*. *Manag*. *Eng*. 2014;8(5):1348–53.

[18] Jeong C. Marine tourist motivations comparing push and pull factors. *J*. *Qual*. *Assur*. *Hosp*. *Tour*. 2014;15(3):294–309. doi: 10.1080/1528008X.2014.921772

[19] Sastre RP, Phakdee-Auksorn P. Examining tourists’ push and pull travel motivations and behavioral intentions: The case of British outbound tourists to Phuket, Thailand. *J*. *Qual*. *Assur*. *Hosp*. *Tour*. 2017;18(4):437–64. doi: 10.1080/1528008X.2016.1250242

[20] Fieger P, Prayag G, Bruwer J. ‘Pull’motivation: an activity-based typology of international visitors to New Zealand. *Curr*. *Issues Tour*. 2019;22(2):173–96. doi: 10.1080/13683500.2017.1383369

[21] Wen J, Huang S. The effects of push and pull travel motivations, personal values, and destination familiarity on tourist loyalty: A study of Chinese cigar tourists to Cuba. *Asia Pac*. *J*. *Tour*. *Res*. 2019;24(8):805–21. doi: 10.1080/10941665.2019.1635504

[22] Carvache-Franco M, Carvache-Franco W, Carvache-Franco O, Hernández-Lara AB. Analysis of push and pull motivations and the intentions to return and recommend a coastal or marine destination. *J*. *Coas*.*t Res*. 2020;36(6):1313–22. doi: 10.2112/JCOASTRES-D-20-00006.1

[23] Güzel Ö, Sahin I, Ryan C. Push-motivation-based emotional arousal: A research study in a coastal destination. *J*. *Dest*. *Mark*. *Manag*. 2020;16:100428. doi: 10.1016/j.jdmm.2020.100428

[24] Yoon Y, Uysal M. An examination of the effects of motivation and satisfaction on destination loyalty: a structural model. *Tour*. *Manag*. 2005;26(1):45–56. doi: 10.1016/j.tourman.2003.08.016

[25] Assaker G, Vinzi VE, O’Connor P. Examining the effect of novelty seeking, satisfaction, and destination image on tourists’ return pattern: A two factor, non-linear latent growth model. *Tour*. *Manag*. 2011;32(4):890–901. doi: 10.1016/j.tourman.2010.08.004

[26] Jang SS, Feng R. Temporal destination revisit intention: The effects of novelty seeking and satisfaction. *Tour*. *Manag*. 2007;28(2):580–90. doi: 10.1016/j.tourman.2006.04.024

[27] Kim J, Chang M, Kim D. Effects of food involvement and novelty seeking on culinary tourism behavior and intension of revisiting the jeonju bibimbab food festival. *Int*. *J*. *Tour*. *Hosp*. *Res*. 2016;30(6):71–84. doi: 10.1080/10941665.2016.1175488

[28] Lee CK, Reisinger Y, Lee J. Examining visitor motivations for mega-events: Comparison between Shanghai Expo and Yeosu Expo. 관광연구저널. 2015;29(10):5–17.

[29] Huyen KN, Nghi NQ. Impacts of the tourists’ motivation to search for novelty to the satisfaction and loyalty to a destination of Kien Giang marine and coastal adventure tourism. *Int*. *J*. *Soc*. *Sci*. *Econ*. *Res*. 2019; 4(4):2807–18. http://www.ijsser.org/

[30] Alipour H, Olya HGT, Maleki P, Dalir S. Behavioral responses of 3S tourism visitors: Evidence from a Mediterranean Island destination. *Tour*. *Manag*. *Perspect*. 2020;33:100624. doi: 10.1016/j.tmp.2019.100624

[31] Pestana MH, Parreira A, Moutinho L. Motivations, emotions and satisfaction: The keys to a tourism destination choice. *J*. *Dest*. *Mark*. *Manag*. 2019;16. doi: 10.1016/j.jdmm.2018.12.006

[32] Rice J, Khanin D. Why do they keep coming back? The effect of push motives vs. pull motives, and attribute satisfaction on repeat visitation of tourist destinations. *J*. *Qual*. *Assur*. *Hosp*. *Tour*. 2019;20(4):445–69. doi: 10.1080/1528008X.2018.1553117

[33] Fianto AYA. Satisfaction as intervening for the antecedents of intention to revisit: Marine tourism context in East Java. Relasi: *Jurnal Ekonomi*. 2020;16(1):179–207. doi: 10.31967/relasi.v16i1.347

[34] Galápagos National Park. Annual Report 2019. Tourism in Galapagos Islands is recovering. https://galapagos.gob.ec/el-turismo-en-galapagos-se-recupera/

[35] Kim KH, Park DB. Relationships among perceived value, satisfaction, and loyalty: Community-based ecotourism in Korea. *J*. *Travel Tour*. *Mark*. 2017;34(2):171–91. doi: 10.1080/10548408.2016.1156609

[36] Lee TH, Jan FH, Tseng CH, Lin YF. Segmentation by recreation experience in island-based tourism: A case study of Taiwan’s Liuqiu Island. *J*. *Sustain*. *Tour*. 2018;26(3):362–78. doi: 10.1080/09669582.2017.1354865

[37] Kline RB. Principles and Practice of Structural Equation Modeling. 6th ed. New York: The Guilford Press; 2011.

[38] MacCallum RC, Browne MW, Sugawara HM. Power analysis and determination of sample size for covariance structure modeling. Psychol Methods. 1996; 1(2):130–49. https://psycnet.apa.org/buy/1996-04469-002

[39] Hooper D, Coughlan J, Mullen M. Structural Equation Modelling: Guidelines for Determining Model Fit. *Electron*. *J*. *Bus*. *Res*. *Methods*. 2008;6(1):53–60.

[40] Kline RB. Principles and Practice of Structural Equation Modeling. New York: Guilford Press; 2005.

[41] Steiger JH. Understanding the limitations of global fit assessment in structural equation modeling. Pers Individ Dif. 2007;42(5):893–8.

[42] Wu W, West SG, Taylor AB. Evaluating model fit for growth curve models: Integration of fit indices from SEM and MLM frameworks. *Psychol*. *Methods*. 2009;14(3):183. https://psycnet.apa.org/doi/10.1037/a0015858 19719357 10.1037/a0015858

[43] Chung Y, Song T, Park J. Freeway booking policy: Public discourse and acceptability analysis. *Transp*. *Policy* (Oxf). 2012;24:223–31. doi: http%3A//dx.doi.org/10.1016/j.tranpol.2012.08.004

[44] Delbosc A, Currie G. Modelling the causes and impacts of personal safety perceptions on public transport ridership. *Transp*. *Policy* (Oxf). 2012;24:302–9. doi: http%3A//dx.doi.org/10.1016/j.tranpol.2012.09.009

[45] Van Acker V, Witlox F. Car ownership as a mediating variable in car travel behaviour research using a structural equation modelling approach to identify its dual relationship. *J*. *Transp*. *Geogr*. 2010;18(1):65–74. doi: http%3A//dx.doi.org/10.1016/j.jtrangeo.2009.05.006

[46] Fitó-Bertran À, Hernández-Lara AB, López ES. The effect of competences on learning results an educational experience with a business simulator. *Comput*. *Human*. *Behav*. 2015;51:910–4. doi: 10.1016/j.chb.2014.11.003

